# Recent Advance in Single-Molecule Fluorescent Biosensors for Tumor Biomarker Detection

**DOI:** 10.3390/bios14110540

**Published:** 2024-11-07

**Authors:** Jie Zhang, Jiawen Liu, Lixue Qiao, Qian Zhang, Juan Hu, Chun-yang Zhang

**Affiliations:** 1 School of Chemistry and Chemical Engineering, State Key Laboratory of Digital Medical Engineering, Southeast University, Nanjing 211189, Chinazhangcy@seu.edu.cn (C.-y.Z.); 2 College of Chemistry, Chemical Engineering and Materials Science, Shandong Normal University, Jinan 250014, China; 3 School of Chemistry and Materials Science, Ludong University, Yantai 264025, China

**Keywords:** biosensor, biomarker, DNA/RNA sensor, enzymatic biosensor, single-molecule biosensor, fluorescence microscopy

## Abstract

The construction of biosensors for specific, sensitive, and rapid detection of tumor biomarkers significantly contributes to biomedical research and early cancer diagnosis. However, conventional assays often involve large sample consumption and poor sensitivity, limiting their further application in real samples. In recent years, single-molecule biosensing has emerged as a robust tool for detecting and characterizing biomarkers due to its unique advantages including simplicity, low sample consumption, ultra-high sensitivity, and rapid assay time. This review summarizes the recent advances in the construction of single-molecule biosensors for the measurement of various tumor biomarkers, including DNAs, DNA modifications, RNAs, and enzymes. We give a comprehensive review about the working principles and practical applications of these single-molecule biosensors. Additionally, we discuss the challenges and limitations of current single-molecule biosensors, and highlight the future directions.

## 1. Introduction

Biosensors are powerful tools for quantitative analysis of biomarkers in real samples, providing digital data of dynamic physiological processes for basic biological research and clinical practice [1,2,3]. Currently, classical sensing methods (e.g., gel electrophoresis [4], enzyme-linked immunosorbent assay (ELISA) [5], and polymerase chain reaction (PCR) [6]) often involve expensive antibodies, cumbersome procedures, and insufficient sensitivity, limiting their broad applications. Therefore, the development of novel biosensors with high sensitivity and simplicity is urgently needed. The single-molecule method is a highly sensitive technique for detecting and characterizing individual molecules [7]. Conventional single-molecule sensing platforms include plasmonic [8,9], low-background-noise fluorescent microscopy [10], and nano transducers [11], and they are capable of detecting biomarkers at the concentrations of nanomolar or higher levels. By virtue of its high sensitivity and ease of use [12], single-molecule detection technology has been widely applied in the fields of biomedical research [13,14], drug development [15,16], biosensors [17,18], and nanotechnology [19,20].

In recent years, single-molecule fluorescence detection has attracted extensive attention because of its unique advantages including simplicity, low sample consumption, ultra-high sensitivity, and rapid assay time. Single-molecule fluorescence detection enables the precise quantification of targets by directly counting individual fluorescence bursts. These single-molecule fluorescent biosensors exhibit sensitivity and resolution, allowing for measuring biomolecules such as proteins [21], nucleic acids [22,23], and metabolites [24,25]. Herein, we review the working principles and applications of single-molecule fluorescent biosensors for the measurement of various tumor biomarkers, including DNAs, DNA modifications, RNAs, and enzymes.

## 2. Single-Molecule Fluorescent Biosensors

New fluorescence microscopy techniques and experimental methods have evolved over the years for resolving the nanostructures at sub-nanometer in size and biosensing at the single-molecule level. In single-molecule fluorescent biosensors, the signals of biomarkers can be translated to the fluorescence signals by specific in vitro/in vivo fluorescent labeling, and the resultant fluorescence signals can be simply detected by single-molecule fluorescence imaging. Confocal microscopy and total internal reflection fluorescence (TIRF) microscopy are frequently employed to detect various biomarkers. In contrast to the measurement of the ensemble average by the conventional methods, single-molecule fluorescent biosensors enable the real-time detection of individual tumor biomarkers with good specificity and high accuracy. Single-molecule fluorescent biosensors possess distinct characteristics of high sensitivity and low sample consumption. Table 1 shows the comparison of a single-molecule fluorescent biosensor with other biosensors for DNA assay in terms of the limit of detection (LOD) and sample consumption.

Single-molecule fluorescent biosensors are usually developed on the basis of single-molecule fluorescent counting and a single-molecule Förster resonance energy transfer (FRET). For single-molecule fluorescent counting, the tumor biomarkers are labeled by the fluorescent dyes/materials and detected via single-molecule fluorescence imaging [32]. The resultant fluorescent signals indicate the presence of tumor biomarkers, and tumor biomarkers can be quantified by simply counting the individual fluorescence signals. Single-molecule FRET relies on the subnanometer distance change at the single-molecule level [33], and typical donor/acceptor pairs are usually derived from the cyanine family (e.g., the pair of Cy3 and Cy5). Semiconductor quantum dots (QDs) have significant advantages of a high quantum yield, good photostability, long fluorescence lifetime, broad absorption, size-dependent emission spectra with narrow bandwidths, good resistance to photodegradation, and a stable surface for chemical modification [34], and they can act as the donors in a single-molecule FRET as well.

## 3. Biosensing Applications

### 3.1. Detection of DNAs and DNA Modifications

**Figure 1:**
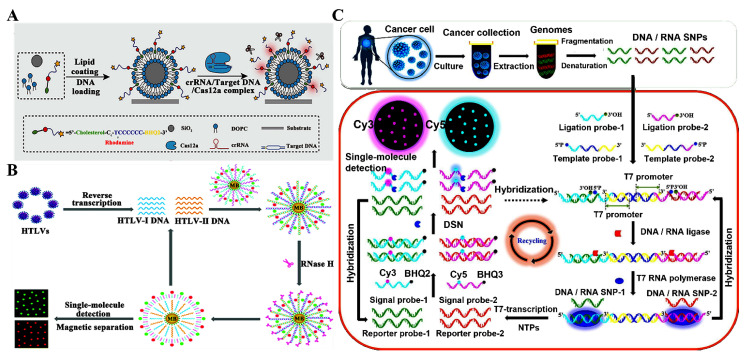
(**A**) CRISPR/Cas12a-integrated single-microbead sensing platform for amplification-free measurement of DNA [26]. (**B**) RNase H-based single-molecule biosensor for simultaneously detecting multiple HTLV DNAs [22]. (**C**) Ligation–transcription circuit-based single-molecule biosensor for simultaneous determination of multiple SNPs [59].

Genomic DNA plays a crucial role in disease risk assessment, early diagnosis, and prognostic monitoring [35,36]. DNA is a key biomarker for disease detection due to its unique characteristics and pivotal role in human biology [37,38]. The development of biosensors capable of detecting specific gene sequences and variants can provide valuable insights into disease diagnosis [39,40], prognosis [41], and treatment [42]. Ma et al. constructed a single-microbead detection platform based on clustered regularly interspaced short palindromic repeats (CRISPR/Cas12a) for an amplification-free and one-step measurement of DNA (Figure 1A) [26]. As depicted in Figure 1A, the microbeads are coated with 1,2-dioleoyl-sn-glycero-3-phosphocholine (DOPC), facilitating the enrichment and immobilization of DNAs. The reporter is modified with a black hole quencher-2 (BHQ2) and a fluorophore (rhodamine), and it can be anchored to the DOPC-coated microbeads (MB@DOPC) via hydrophobic interactions between lipids and cholesterols. Target DNA is complimentary to the CRISPR RNA (crRNA) to activate Cas12a, inducing the cyclical cleavage of reporters on the surface of the microbeads and the recovery of fluorescence. The assay is capable of detecting as low as three copies of the target in 5 μL of a sample. In addition, it can identify DNA mutations in real samples without the need for additional nucleic acid amplification.

Virus-related infectious diseases can cause a worldwide public health crisis [43,44]. Human T-cell lymphotropic viruses (HTLVs) belong to the oncovirus subfamily of retroviridae and are closely associated with diverse human diseases [45]. The construction of biosensors for the simultaneous profiling of multiple HTLVs not only contributes to the understanding of the molecular mechanisms of pathogenicity among various subtypes [46] but also facilitates disease diagnosis and prevention [47]. Zhang et al. proposed a ribonuclease H (RNase H)-based single-molecule biosensor for the simultaneous detection of multiple HTLV DNAs in cancer cells [22]. As shown in Figure 1B, targets can bind with the RNA signal probe modified with indodicarbocyanine (Cy5) and indocarbocyanine (Cy3) on the surface of magnetic beads, forming the DNA/RNA heteroduplexes. Subsequently, RNase H can cleave the phosphodiester bond of RNA signal probes in the heteroduplexes, liberating the Cy5 and Cy3 molecules from the magnetic beads into the solution and releasing target DNAs. The released target DNAs pair with new RNA signal probes to trigger the next round of cleavage reaction, inducing the exponential accumulation of Cy5 and Cy3 fluorophores into the solution after magnetic separation, with the signal of Cy3 indicating HTLV-I DNA and Cy5 indicating HTLV-II DNA. This biosensor demonstrates high sensitivity with a limit of detection (LOD) of 82.8 aM for HTLV-II DNA and 66.1 aM for HTLV-I DNA. In addition, it is capable of simultaneously quantifying HTLV-II DNA and HTLV-I DNA at the single-cell level as well as distinguishing HTLV-positive from HTLV-negative cells.

Single nucleotide polymorphisms (SNPs) are DNA sequence variations primarily caused by single nucleotide changes at the genomic level [48], and serve as a significant source of genetic diversity [49,50]. Some SNPs have been shown to be associated with many human diseases, such as multiple sclerosis [51], cystic fibrosis [52], hypertension [53,54], and various cancers [55,56,57]. Therefore, SNPs are considered as promising biomarkers for diagnostic and therapeutic evaluation [58]. Zhang et al. reported a multiple ligation-driven symmetric T7-transcription circuit for the simultaneous analysis of multiple SNPs at the single-molecule level (Figure 1C) [59]. Template probe-2 (TP-2) pairs with template probe-1 (TP-1) to create a TP-1-TP-2 duplex containing partially complementary sequences of BRAF-V600E and KRAS-135C. When BRAF-V600E and KRAS-135C are present, TP-1-TP-2 duplex, ligation probe-1 (LP-1) and ligation probe-2 (LP-2) are ligated together by ligase to generate transcription templates 2 and 1. Subsequently, symmetric transcription amplifications are activated to transcribe two reporter probes (i.e., RP-2 and RP-1). RP-2 and RP-1 can bind with signal probe-2 (SP-2) and signal probe-1 (SP-1), respectively, to initiate the cyclic digestion of SP-2 and SP-1, leading to the recovery of Cy3 and Cy5 fluorescence. Moreover, free RP-2 and RP-1 can act as the ligation templates to initiate the next rounds of ligation–transcription–digestion, leading to the restoration of the Cy3 and Cy5 signal. In addition, this method can accurately distinguish SNPs with sequence homology, differentiate the levels of SNPs in tumor cells and healthy cells, and analyze SNPs in diverse tumor cells at the single-cell level.

**Figure 2:**
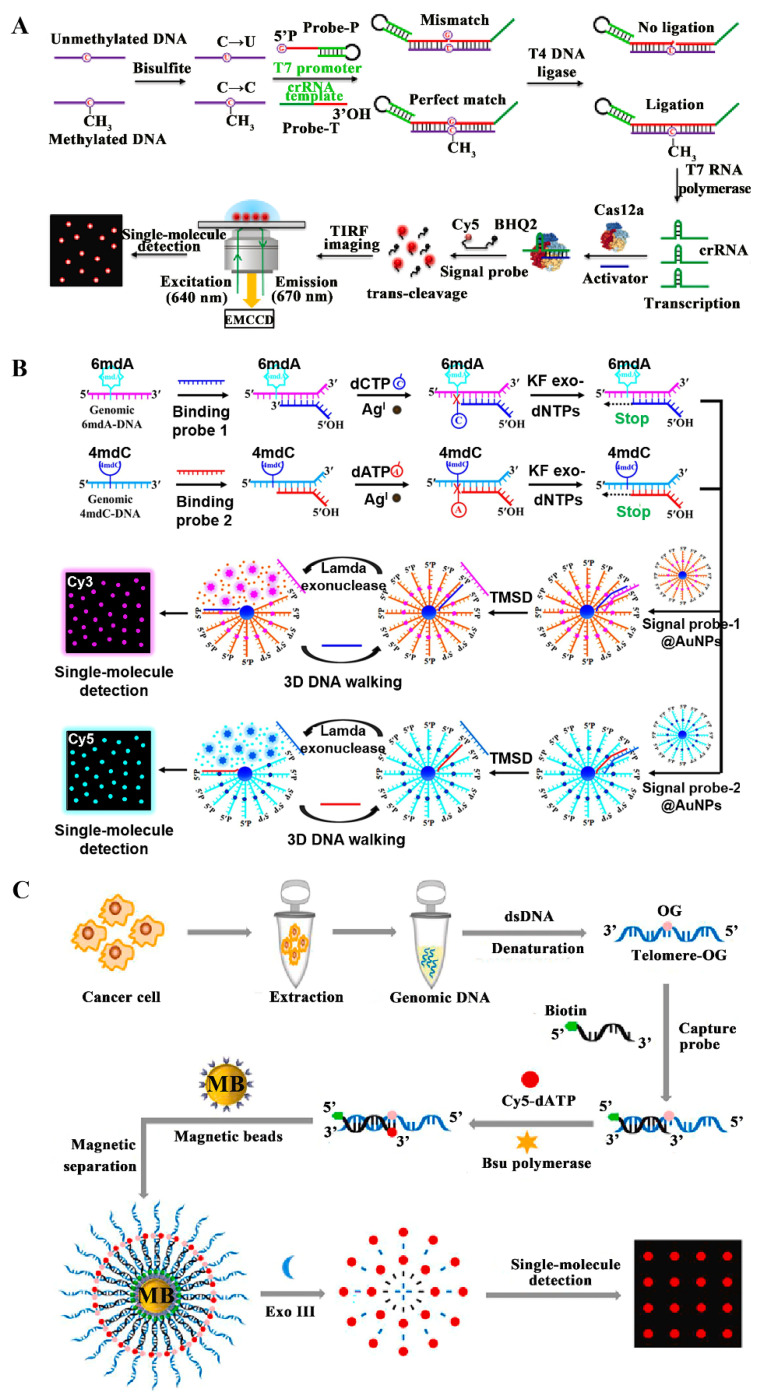
(**A**) Methylation-sensitive transcription-based single-molecule biosensor for accurate analysis of DNA methylation [65]. (**B**) Silver-coordinated Watson–Crick pairing-based single-molecule biosensor for simultaneously detecting genomic 4mdC and 6mdA [71]. (**C**) Bsu DNA polymerase-mediated single-molecule biosensor for rapid detection of OG in telomeres of cancer cells [81].

DNA methylation is a key epigenetic modification in mammals and plays a critical role in regulating cell growth [60], gene expression [61], and the onset of genetic diseases [62]. Abnormal DNA methylation patterns associated with tumor-related genes can contribute to the development and progression of malignant tumors [63,64]. Consequently, DNA methylation has become a potential biomarker for the early detection and prognosis of cancers. Zhang et al. demonstrated a methylation-sensitive transcription-based fluorescent biosensor for a single-molecule quantification of DNA methylation (Figure 2A) [65]. Target methylated DNA remains unchanged upon the treatment with bisulfite. Then, the probe-T and the probe-P can adjacently hybridize with target methylated DNA, initiating the ligation of probe-T catalyzed and probe-P by DNA ligase to produce a full-length transcription substrate. Upon the introduction of NTP mixtures, T7 RNA polymerase recognizes the full-length transcription substrate and transcribes abundant crRNA strands. The synthesized crRNAs can pair with single-stranded DNA (ssDNA) activators to activate Cas12a, inducing the cyclic degradation of quencher and fluorophore dual-labeled signal probes and eventually generating the amplified fluorescence signals. This single-molecule biosensor is able to distinguish as low as 0.01% methylation level, and even quantify genomic DNA methylation in a single cancer cell and clinical samples, offering a powerful tool for early diagnosis and epigenetic studies.

Both N6-methyladenine (6mdA) and N4-methylcytosine (4mdC) are common DNA modifications in prokaryotes and eukaryotes [66,67,68]. The development of efficient methods that can simultaneously and selectively monitor both 4mdC and 6mdA at specific sites is important for epigenetic research and clinical disease diagnosis. However, the conventional hybridization-based techniques are not applicable for 6mdA/4mdC assays [69,70]. Zhang et al. developed a silver-coordinated Watson–Crick pairing-based single-molecule biosensor for simultaneously detecting genomic 6mdA and 4mdC [71]. As illustrated in Figure 2B, 4mdC-DNA and 6mdA-DNA can pair with binding probes 2 and 1, respectively, leading to the generation of 4mdC-DNA-BP2 and 6mdA-DNA-BP1 duplexes. In the formed 4mdC-DNA-BP2 and 6mdA-DNA-BP1 duplexes, binding probes 1 and 2 cannot be extended by Klenow fragment (3’→5’ exo-) DNA polymerase because the 4mdC-A and 6mdA-C mismatches cannot be stabilized by Ag^I^. The signal probes 1/2 immobilized on the gold nanoparticle (AuNP) can hybridize with binding probes 1/2 via toe-mediated strand displacement (TMSD), forming signal probe 1–binding probe 1 and signal probe 2–binding probe 2 duplexes, respectively. Subsequently, lambda exonuclease cyclically cleaves the signal probes of signal probe–binding probe duplexes, inducing the liberation of a large number of Cy3 and Cy5 molecules from the AuNP surface. This single-molecule biosensor displays high sensitivity with an LOD of 9.97 fM for 4mdC and 9.80 fM for 6mdA. This single-molecule biosensor is capable of distinguishing modified DNA from unmodified genomic DNA and detecting modified DNA in specific genomic loci of malignant tumor cells and E. coli plasmid cloning vectors.

8-oxo-7,8-dihydroguanine (OG) is a significant DNA damage marker [72] induced by oxidative stress, such as ionizing radiation, environmental pollutants, and reactive oxygen species [73,74,75,76]. The presence of OG may lead to mutations in the DNA sequence, interfering with the normal process of DNA replication and gene expression and eventually inducing tumor formation [77,78]. Therefore, OG is regarded as a key biomarker for studying DNA damage repair and early diagnosis of oxidative damage-related diseases [72,79,80]. Zhang et al. reported a Bsu DNA polymerase-mediated single-molecule biosensor for a simple and rapid quantification of OG in telomeres of cancer cells (Figure 2C) [81]. The target DNA sequence specifically hybridizes with the biotinylated capture probe to form a 5’-biotinylated DNA duplex. Subsequently, Cy5-dATP is polymerized to the OG opposite site of the capture probe catalyzed by Bsu DNA polymerase, inducing the production of Cy5/biotin-labeled double-stranded DNA (dsDNA). Cy5/biotin-labeled dsDNAs are then self-assembled onto magnetic beads’ surface to form the magnetic bead–dsDNA-Cy5 complexes. After magnetic separation, the dsDNAs on the magnetic beads’ surface are degraded by exonuclease III, releasing a large number of Cy5 fluorophores. This single-molecule biosensor can rapidly measure OG (within 70 min) and achieve an LOD of 2.45 × 10^−18^ M. In addition, this biosensor is able to accurately measure OG in genomic DNA extracted from H_2_O_2_-stimulated cancer cells, showing great promise for early clinical diagnosis and disease-specific genetic damage studies.

### 3.2. Detection of RNAs

**Figure 3:**
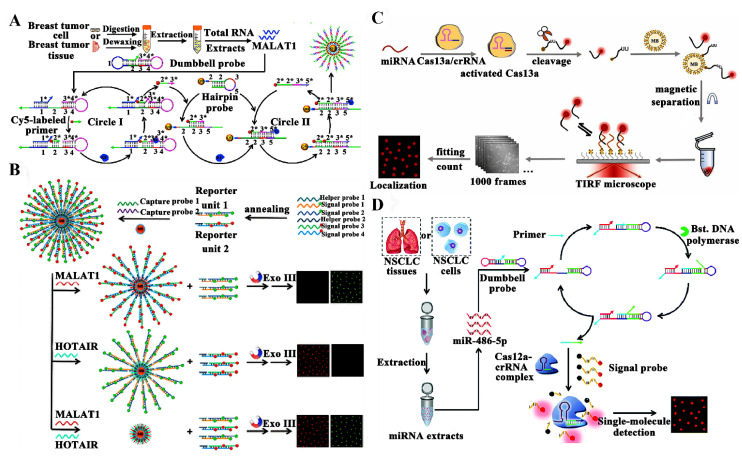
(**A**) QD-based single-molecule biosensor for monitoring lncRNAs by coupling PER with isothermal circular strand-displacement polymerization reaction [102]. (**B**) Single-molecule biosensor for the simultaneous quantification of multiple lncRNAs by utilizing magnetic separation techniques and enzyme-free strand displacement reaction [89]. (**C**) Single-molecule biosensor for quantitative analysis of miRNA assay by integrating CRISPR/Cas13a with DNA-PAINT [100]. (**D**) Single-molecule biosensor for one-step detection of miRNAs in lung cancer tissues by utilizing PER and CRISPR-Cas12a [101].

Long non-coding RNAs (lncRNAs) are a class of RNA molecules that lack protein-coding capacity with more than 200 nucleotides in length [82]. LncRNAs play crucial roles in gene regulation, including transcriptional [83] and post-transcriptional regulation [84], and epigenetic modulation [85]. The dysregulation of lncRNAs is implicated in a wide range of diseases, including cancer, making them become important biomarkers in diagnostics and therapeutics [86,87,88]. Zhang et al. proposed a quantum dot (QD)-based single-molecule biosensor for monitoring lncRNAs by coupling the primer exchange reaction (PER) with the isothermal circular strand-displacement polymerization reaction. As presented in Figure 3A, target lncRNA can open the left hairpin structure of the dumbbell probe through a hybridization reaction, leading to the exposure of the binding region of the Cy5-labeled primer. The Cy5-labeled primer then hybridizes with the exposed binding region of the dumbbell probe and is extended with the help of Klenow fragment DNA polymerase to produce the Cy5-labeled initiator. Afterwards, the complementary domain of the amplification region of the dumbbell probe can competitively substitute the extending region of the Cy5-labeled initiator, inducing the liberation of the Cy5-labeled initiator. The new Cy5-labeled primer can in turn bind with the unlocked dumbbell probe to initiate a new PER, generating a large number of Cy5-labeled initiators. The biotinylated hairpin probes on the 605QD surface are opened by the Cy5-labeled initiators through a hybridization reaction, activating an isothermal circular strand-displacement polymerization reaction to generate multitudinous 605QD-dsDNA-Cy5 nanostructures. This biosensor only uses one DNA polymerase to achieve high sensitivity with an LOD of 65.25 aM, and it is applicable for distinguishing tumor cells from normal cells, measuring the lncRNA concentrations at the single-cell level, and differentiating telomerase activity between normal and cancer cells.

Notably, the above single-molecule sensing methods are only suitable for detecting single lncRNA. The construction of single-molecule sensing strategies for the simultaneous determination of multiple lncRNAs is urgently needed. Zhang et al. developed a single-molecule biosensor for the simultaneous quantification of multiple lncRNAs by utilizing magnetic separation techniques and an enzyme-free strand displacement reaction (Figure 3B) [89]. MB-capture probe-multiple Cy5/Cy3-modified reporter unit complexes are carefully designed for the recognition and detection of two target lncRNAs. LncRNA MALAT1 and HOTAIR can hybridize with capture probes 1 and 2, respectively, resulting in the dissociation of reporter units 1 and 2 from the surface of MB. After magnetic separation, free reporter units 1 and 2 are digested into single nucleotides by exonuclease III, producing high fluorescence signals. This biosensor displays high sensitivity with an LOD of 0.031 aM for lncRNA HOTAIR and 0.10 aM for lncRNA MALAT1. Additionally, it is able to distinguish cancer cells from healthy cells and image intracellular lncRNAs.

MicroRNAs (miRNAs) are defined as small non-coding RNA molecules with 21–25 nucleotides in length, and participate in genes’ expression involved in diverse biological processes (e.g., cell differentiation, proliferation, development, and apoptosis) [90,91,92,93,94,95]. Disregulated miRNA expression is linked to a diversity of diseases, including neurological conditions, cardiovascular disorders, and cancers, making miRNAs promising targets for therapeutic intervention and diagnostic biomarkers [96,97,98,99]. Hao et al. constructed a single-molecule biosensor for a sensitive and simple analysis of miRNA by combining the CRISPR/Cas13a system with DNA point accumulation in nanoscale topology (DNA-PAINT) [100]. As illustrated in Figure 3C, target schistosome-derived miRNA (sja-miR-2c-5p) can be specifically recognized by the crRNA to activate the RNase activity of Cas13a, indiscriminately cleaving fluorescent probes. Then, magnetic beads are added into the amplification system to remove the uncleaved fluorescent probes. The cleaved probes can bind to the capture probes (CPs) immobilized on the coverslip surface, forming a “binding-unbinding” dynamic equilibrium. This single-molecule biosensor achieves a low LOD of 1.12 fM, and possesses the capability of measuring miRNAs in complex biological samples.

Alternatively, Zhang et al. proposed a single-molecule biosensor for a one-step detection of miRNAs in lung cancer tissues by utilizing the PER and CRISPR-Cas12a system [101]. As presented in Figure 3D, they designed a dual-functional dumbbell probe as both the detection probe for target miRNA recognition and the template for PER amplification. Target miRNA can hybridize with the dumbbell probe, causing the exposure of the complementary domain of the PER primer. Subsequently, PER amplification is activated to produce a large number of extended primers. The extended primer then binds with crRNA to activate Cas12a, inducing the cyclic cleavage of signal probes and the recovery of Cy5 fluorescence. This assay is very simple and it can be completed in a one-step reaction without the involvement of tedious separation/washing steps and precise temperature control. Moreover, this assay possesses high sensitivity, and it can discriminate single-base mismatch. Furthermore, it can accurately measure the expression level of miR-486-5p in various types of lung cancer cells, and can even distinguish non-small cell lung cancer (NSCLC) patients from healthy persons.

**Figure 4:**
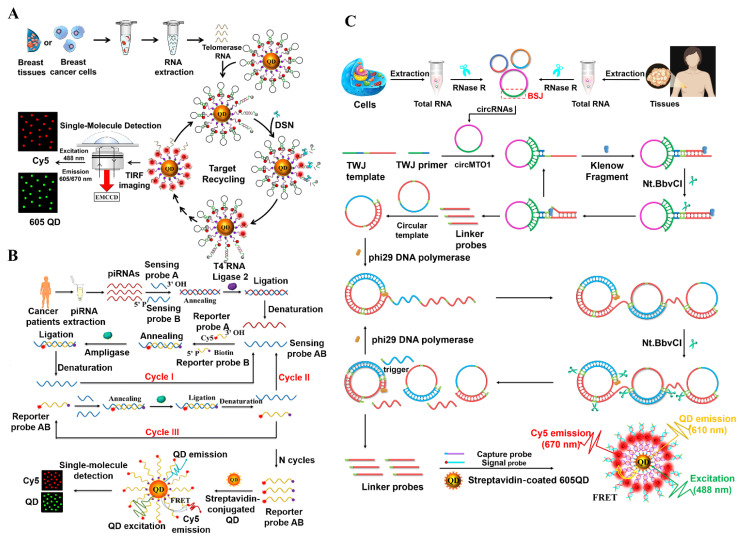
(**A**) DSN-driven QD biosensor for one-step measurement of hTR at the single-molecule level [106]. (**B**) QD biosensor based on multi-cycle ligation amplification for sensitive detection of piRNA [111]. (**C**) QD biosensor for highly specific detection of circRNA based on TWJ skeleton and EXP-RCA [120].

Human telomerase RNA (hTR) is a key component of telomerase and it can indirectly reflect the telomerase activity [103,104]. The expression of hTR is closely associated with tumor grading [105], and thus, the accurate measurement of hTR is important for the diagnosis and treatment of telomerase-targeted tumors. Zhang et al. developed a double-stranded specific nuclease (DSN)-driven QD biosensor for a one-step measurement of hTR at the single-molecule level [106]. As displayed in Figure 4A, a biotinylated hairpin probe is modified with Cy5 and BHQ2, and it has the functions of target recognition, signal amplification, and readout. In the 605QD-Cy5 probe-BHQ2 complex, the Cy5 emission induced by the FRET from 605QD to Cy5 is quenched by the nearby BHQ2. Target hTR can be specifically captured by the hairpin probe on the QD surface to initiate a DSN-mediated DNA-specific cleavage reaction, leading to the dissociation of the BHQ2 molecule from the 605QD-ssDNA-Cy5 structure and restoration of the Cy5 fluorescence signal. Meanwhile, the hTR is separated from the cleaved hairpin probe, initiating the next round of hybridization–cleavage reactions to generate the amplified Cy5 fluorescence signals. The biosensor has the advantages of simplicity, rapidity (analysis time of 60 min), and high sensitivity (detection limit of 2.10 fM). In addition, it allows the effective differentiation of single-base mismatch sequences and precise distinguishing of hTR levels between healthy donors and cancer patients.

Piwi-interacting RNAs (piRNAs) are a novel category of small non-coding RNAs with high stability, and they can be detected in human body fluids [107,108]. The abnormal expression of piRNA is a feature of multiple tumor types [109,110]. Consequently, accurate quantification of piRNA is of great value for basic research and cancer diagnosis. Zhang et al. constructed a QD biosensor based on multi-cycle ligation amplification for sensitively detecting piRNA [111]. As illustrated in Figure 4B, when target piRNA is present, sensing probes B and A hybridize adjacently with piRNA to synthesize the sensing probe AB with the aid of T4 RNA ligase. After thermal denaturation, the sensing probe AB can hybridize with biotin-modified reporter probe B and Cy5-modified reporter probe A, initiating DNA ligase-mediated ligation-denaturation amplification (Cycle I) to produce numerous reporter probes AB. The obtained reporter probes AB subsequently hybridize with sensing probes B and A to activate their ligation, resulting in the generation of sensing probes AB. The ligated sensing probes AB can be used as the templates to initiate the cyclic ligation of reporter probes B and A (Cycle II), resulting in the generation of a large number of reporter probes AB. In the meantime, the reporter probes AB can function as the ligation templates for sensing probes B and A to trigger a cycle of hybridization–ligation–denaturation (Cycle III), yielding abundant sensing probes AB. The Cy5- and biotin-labeled reporter probes AB can be anchored to the 605QD surface to obtain a 605QD/reporter probe AB/Cy5 nanocomplex, generating efficient FRET signals. This biosensor is capable of sensitively quantifying piRNAs in different cell lines and distinguishing piRNA expression in cancer patients and healthy paracancerous tissues, providing a reliable platform for piRNA-associated disease diagnosis and biomedical research.

Circular RNAs (circRNAs) are a novel type of non-coding RNAs produced by non-canonical backsplicing with high conservation and stability [112,113]. CircRNAs are involved in a wide variety of cellular physiological processes, such as mRNA sequestration, gene transcription and translation, RNA splicing, and protein localization [114,115]. The dysregulated expression of circRNAs has been identified as potential oncogenes, and it is strongly involved in the progression of various human malignancies, such as the liver [116], lung [117], colorectal [118], and breast cancers [119]. Consequently, circRNAs may serve as the diagnosis biomarkers for circRNAs-associated diseases. Taking advantage of the three-way junction (TWJ) skeleton and exponential-rolling circle amplification (EXP-RCA), Zhang et al. constructed a QD biosensor for a highly specific analysis of circRNA [120]. As displayed in Figure 4C, the circRNA mitochondrial tRNA translation optimization 1 (circMTO1) can hybridize with the TWJ primer and TWJ template to create a TWJ skeleton. In the formed TWJ skeleton, the TWJ primer can be extended by DNA polymerase to initiate the cyclic strand displacement reaction with the aid of Nt.BbvCI, producing abundant linker probes. The polymerized linker probe is complementary to a circular template and can act as a primer to initiate an RCA reaction, inducing the synthesis of long ssDNAs with repetitive sequences. Long ssDNA products can be recognized and cleaved into multiple short ssDNA strands by Nt.BbvCI. The obtained ssDNA strands can act as the primers to bind with the circular templates, initiating the RCA reaction to generate a large number of linker probes. Afterwards, the resulting linker probes can serve as a “bridge” to connect the Cy5-modified signal probe and the biotinylated capture probe, producing sandwich duplexes that can self-assemble onto the QD surface. This QD biosensor has the advantages of a near-zero background signal, single base mismatch specificity, high amplification efficiency, and eliminating the need for exogenous reverse transcription primers. It can accurately measure the circRNA levels with single-cell sensitivity, and distinguish the circRNA levels in breast cancerous tissues and adjacent normal tissues.

### 3.3. Detection of Enzymes

**Figure 5:**
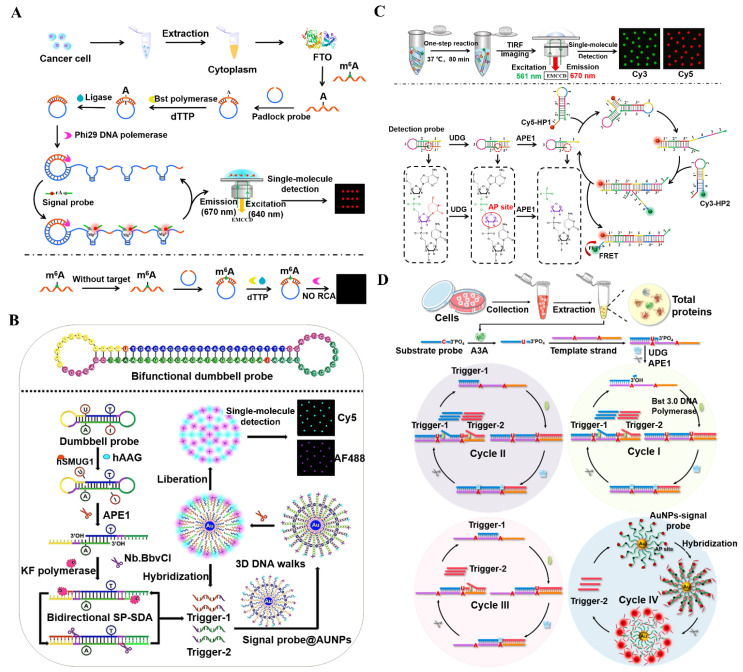
(**A**) Multiple DNAzyme-powered biosensor for single-molecule monitoring of FTO [127]. (**B**) Bidirectional strand displacement-driven single-molecule biosensor for simultaneously measuring hAAG and hSMUG1 [128]. (**C**) Single-molecule biosensor for the measurement and imaging of UDG activity based on CHA-induced FRET [138]. (**D**) AuNP-based single-molecule biosensor for monitoring APOBEC3A activity by utilizing ERA reaction [144].

The fat mass and obesity-associated protein (FTO) is a kind of RNA demethylase and it removes methyl groups from the N^6^-methyladenosine (m^6^A) located in RNA [121]. The demethylation activity of FTO is implicated in various biological processes (e.g., metabolism [122], development [123], and neural function [124]), and its dysregulated expression may interfere with the normal demethylation process, ultimately leading to various human diseases [124,125,126]. Consequently, a sensitive measurement of FTO activity is highly valuable for anticancer drug discovery and clinical diagnostics. Zhang et al. developed a multiple DNAzymes-based biosensor for a single-molecule monitoring of FTO (Figure 5A) [127]. In the presence of FTO, the m^6^A-RNA probe is demethylated to generate an A-RNA probe. The A-RNA probe is then used as a template to pair with the padlock probe, activating a polymerization–ligation amplification in the presence of DNA polymerase and ligase to generate a closed circular template. Subsequently, polymerase-mediated RCA is triggered to yield a long ssDNA product containing Mg^2+^-dependent DNAzyme repeats. The synthesized DNAzymes can cyclically degrade the signal probes to release multiple Cy5 molecules. This assay shows high sensitivity and good selectivity, and it can be utilized for the screening of FTO inhibitors and quantification of the FTO level at the single-cell level.

An accurate and simultaneous measurement of multiple DNA glycosylases is essential for the diagnosis of DNA glycosylase-related diseases. Zhang et al. designed a bidirectional strand displacement-driven single-molecule biosensor for simultaneously measuring human alkyladenine glycosylase (hAAG) and human single-stranded-selective monofunctional uracil glycosylase (hSMUG1) [128]. As illustrated in Figure 5B, this biosensor only employs a dumbbell probe and two Cy5- and Alexa Fluor 488-modified signal probes for two DNA glycosylases assay. HSMUG1 and hAAG can remove deoxyuridine and deoxyinosine bases from the dumbbell probe, respectively, generating two apurinic/apyrimidinic (AP) sites. Upon the addition of APE1, the obtained AP sites of the dumbbell probe are hydrolyzed to unfold the dumbbell probe, accompanied by the generation of two 3′-OH ends. Subsequently, the bidirectional strand displacement reaction is initiated under the coexistence of DNA polymerase and Nb.BbvCI, leading to the generation of a large number of triggers 1 and 2. The generated triggers can bind to the signal probes on the AuNP surface to activate an APE1-mediated hybridization–cleavage reaction circuit, liberating large amounts of Cy5 and Alexa Fluor 488 molecules. This biosensor displays ultrahigh sensitivity with an LOD of 4.50 × 10^−9^ U/μL for hAAG and 8.14 × 10^−10^ U/μL for hSMUG1. It can further identify the inhibitors, measure DNA glycosylases’ activity in a single cancer cell, and differentiate glycosylases in cancer and normal cells.

DNA glycosylases are a class of initiating enzymes involved in the base excision repair (BER) process, and they can recognize/excise damaged or inappropriate bases from DNA [129,130,131,132,133,134,135]. DNA glycosylase is involved in diversified cellular physiological processes, and its dysregulated expression may impair the BER pathway, inducing a variety of human diseases (e.g., malignant tumors and neurodegenerative diseases) [136,137]. Zhang et al. proposed a single-molecule biosensor for the quantitation and imaging of uracil-DNA glycosylase (UDG) activity based on catalyzed hairpin assembly (CHA)-induced FRET (Figure 5C) [138]. UDG can remove the uracil base of the detection probe to form a single nucleotide gap with the aid of apurinic/apyrimidinic endonuclease (APE1), exposing the toehold domain of the detection probe. Afterwards, the cleaved detection probe acts as a catalyst to cyclically initiate the CHA reaction between Cy5-HP1 and Cy3-HP2, inducing the FRET from Cy3 to Cy5 and consequently the decrease in the Cy3 signal and the increase in the Cy5 signal. This single-molecule biosensor can be rapidly accomplished in an enzyme-free manner, simplifying the reaction procedure and making it suitable for cellular imaging. This biosensor can be utilized to determine kinetic parameters and distinguish the UDG levels in HeLa cells and HL-7702 cells.

APOBEC3A is an important member of the APOBEC (apolipoprotein B mRNA editing catalytic polypeptide-like) family, and it is a cytidine deaminase with a crucial role in DNA repair and immune responses within cells (e.g., inhibition of viral replication and restriction of exogenous DNA integration) [139,140,141]. APOBEC3A has been implicated in multiple cancers [142,143]. Zhang et al. demonstrated a AuNP-based single-molecule biosensor for monitoring APOBEC3A activity by utilizing enzymatic repairing amplification (ERA) [144]. As illustrated in Figure 5D, when target APOBEC3A is present, the cytosine of the substrate probe is specifically converted to uracil, and then the deaminated substrate probe is paired with the template strand to obtain the dsDNA containing U/A base pairs. In the formed substrate probe-template strand dsDNA, the U base is excised by UDG to generate multiple single nucleotide gaps with the assistance of APE1. Upon the addition of DNA polymerase, the cyclic ERA reaction is activated, leading to the exponential accumulation of trigger 2. The generated trigger 2 can bind to the signal probe immobilized on the AuNP surface to activate the APE1-catalyzed cyclic digestion of signal probes, inducing the release of multitudinous Cy5 fluorophores. This biosensor is capable of selectively and sensitively detecting the APOBEC3A activity with an LOD of 0.855 aM. Moreover, it can be used for the measurement of kinetic parameters, screening of APOBEC3A inhibitors, and quantification of APOBEC3A activity at the single-cell level, with promising applications in cancer diagnosis.

**Figure 6:**
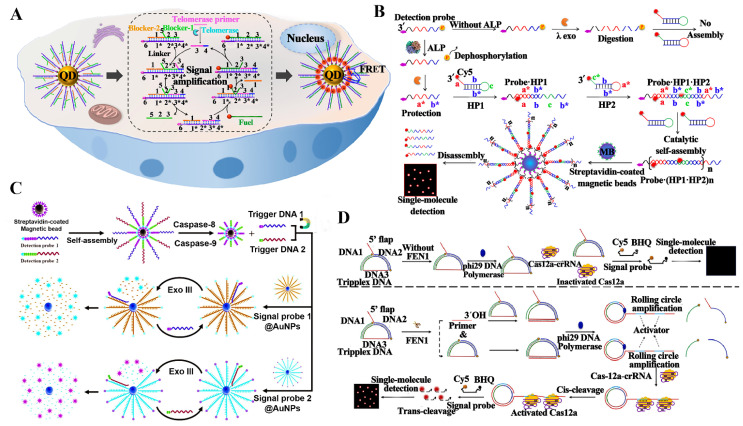
(**A**) Enzyme-free entropy-driven QD biosensor for the imaging of intracellular telomerase activity [145]. (**B**) HCR-based biosensor for single-molecule monitoring of ALP [156]. (**C**) AuNP-based nanomachine for the quantification of caspase-8 and caspase-9 by combining Exo III-mediated signal amplification with single-molecule detection [166]. (**D**) RCA-integrated CRISPR/Cas12a biosensor for isothermal single-molecule measurement of FEN1 in breast cancer tissues [173].

Telomerase is a ribonucleoprotein enzyme complex, and it can add telomeric repeat sequences (“TTAGGG” in vertebrates) to the ends of chromosomal DNA for maintaining the integrity of eukaryotic chromosomes [145,146]. The dysregulation of telomerase activity has been implicated in different kinds of age-related diseases (e.g., certain genetic disorders and multiple malignant tumors) [147,148,149]. Zhang et al. developed an enzyme-free entropy-driven QD biosensor for the imaging of intracellular telomerase activity (Figure 6A) [145]. Telomerase can specifically recognize and extend the telomerase primer, resulting in the generation of a complete toehold at the 3’-end of the telomerase primer. Afterwards, the extended telomerase primer can act as an initiator to launch an entropy-driven catalytic (EDC) circuit, producing numerous biotin- and Cy5-modified duplexes. The resulting duplexes can rapidly anchor to the 605QD surface to shorten the distance between the Cy5 acceptor and the 605QD donor, inducing a significant FRET from 605QD to Cy5. The whole reaction may be completed in one step under a constant temperature, eliminating strict temperature control and complicated reaction procedures and greatly shortening the assay time. The QD biosensor allows the precise quantification of telomerase activity with single-cell sensitivity, and may be used for the imaging telomerase in living cells, holding promising potential in telomerase-based cancer diagnosis and therapy.

Alkaline phosphatase (ALP) is an enzyme that hydrolyzes phosphate ester bonds, and it is present in a wide variety of organisms including humans, animals, and plants [150,151,152]. In clinical diagnostics, the ALP levels are commonly used to assess liver [153], bone [154], and biliary tract diseases [155]. ALP is also a biomarker used to evaluate bone metabolism [151] and liver function [155]. By coupling the hybridization chain reaction (HCR) with single-molecule detection technology, Zhang et al. constructed a simple fluorescence biosensor for the monitoring of ALP [156]. As presented in Figure 6B, the 5’ phosphate group-modified detection probe is dephosphorylated by ALP, which can effectively protect the detection probe from lambda exonuclease-induced digestion. The intact detection probe then acts as a trigger to activate the HCR amplification between Cy5-modified HP2 and Cy5-modified HP1, forming a long double-stranded DNA detection probe-(HP1-HP2)_n_. Biotin-modified long fluorescent dsDNAs can rapidly assemble onto the magnetic beads’ surface, and are subsequently separated from the remaining HP2 and HP1 with the aid of a magnet. Upon the introduction of NaOH, the purified long fluorescent dsDNAs are broken down into free HP2 and HP1. This biosensor exhibits an LOD of 2.61 × 10^−6^ U mL^−1^, and it is suitable for assessing the ALP inhibitor and quantifying the cellular ALP activity with single-cell sensitivity.

Caspases are a family of protease enzymes crucial for programmed cell death and apoptosis in multicellular organisms [157]. Caspases play key roles in regulating multifarious cellular processes, including cell differentiation [158], proliferation [159], and immune responses [160,161]. An imbalance of caspases’ expression is linked to many diseases, including cancer, neurodegenerative disorders, and autoimmune diseases [162,163,164,165]. Zhang et al. proposed a AuNP-based nanomachine for the quantification of caspase-8 and caspase-9 by combining Exo III-mediated signal amplification with a single-molecule detection [166]. The designed sensing strategy involves two functional particles, including peptide-DNA detection probes 1/2-modified magnetic beads and signal probes 1/2-modified AuNPs (signal probes@AuNPs). As displayed in Figure 6C, caspase-9 and caspase-8 specifically recognize and hydrolyze the peptide bond of detection probes 2 and 1, inducing the release of cleaved detection probes 1 and 2 from magnetic beads, respectively. The cleaved detection probes 2 and 1 can act as walker DNA to trigger the Exo III-mediated cyclic cleavage of signal probes@AuNPs, liberating numerous Texas Red and Cy5 molecules. This nanomachine displays an LOD of 1.71 × 10^−6^ U/μL for caspase-9 and 2.08 × 10^−6^ U/μL for caspase-8, and it can screen caspase inhibitors and measure cellular caspases’ activity in a single cancer cell.

Flap Endonuclease 1 (FEN1) is a structure-specific endonuclease, and it can cleave the 5′ overhangs of DNA flap structures during DNA BER pathways [167,168]. FEN1 plays a vital role in maintaining genome stability [169,170] and cell cycle regulation [171,172], and it may function as a diagnostic biomarker for various cancers (e.g., ovarian cancer and hepatocellular carcinoma) [137]. Zhang et al. developed an RCA-integrated CRISPR/Cas12a biosensor for an isothermal single-molecule measurement of FEN1 in breast cancer tissues [173]. As displayed in Figure 6D, FEN1 can identify and cleave the flap structure of triplex DNA to release a primer with a free 3’-OH end. Then, the generated primer can perfectly hybridize with the circular template to initiate an RCA reaction, inducing the synthesis of a long ssDNA strand with multiple repeated activator sequences. The synthesized ssDNA product can activate CRISPR/Cas12a, inducing the cyclic degradation of Cy5- and BHQ-labeled signal probes and the recovery of the Cy5 signal. This biosensor is able to detect endogenous FEN1 activity in a single cell, and even distinguish the FEN1 level in healthy paracancerous tissues and breast cancer tissues.

## 4. Conclusions

Single-molecule detection has gained popularity in clinical research and early diagnostics due to its outstanding advantages of high sensitivity, low-volume sample requirement, and high signal-to-noise ratio. In this review, we briefly discussed the application of various single molecule biosensors for detecting disease biomarkers such as DNA [22,26], DNA methylation [65,71], SNP [59], lncRNAs [89,102], microRNA [100,101], and enzymes [127,128,138,144,145,156,166,173]. These single-molecule biosensors exhibit the benefits of high sensitivity and simplicity, and exhibit excellent performance even in complex biological matrices (Table 1), with potential applications in clinical diagnosis and drug discovery. The integration of single-molecule fluorescent detection with nucleic acid amplification can achieve ultrasensitive detection with a wide linear range and a low LOD, facilitating the detection of low-abundance disease biomarkers in clinical samples (e.g., serum and tissues) (Table 2). Despite the significant advantages of single-molecule fluorescent biosensors, several challenges remain to be addressed. (1) Fluorescence microscopes for single-molecule detection are typically complex, bulky, and expensive. Therefore, more effort should be put into developing more affordable and miniaturized devices. By guiding the illumination light through optical fibers and waveguides, a low-cost optical microscope can be obtained to acquire super-resolved images [174]. (2) Only parts of nanomaterials and labeling strategies have been exploited in single-molecule fluorescent biosensors. Great efforts should be put into finding more suitable functional nanomaterials for the construction of single-molecule fluorescent biosensors (e.g., graphene quantum dots as the fluorescent labels, graphene oxides as the fluorescent quenchers, and metal–organic frameworks as the separators and probe carriers). It should be noted that the discovery of new labeling strategies and new fluorescent biomarkers will facilitate the maintenance of high stable performance of single-molecule biosensors under harsh sample conditions. (3) Current single-molecule fluorescence counting is highly dependent on frame-by-frame counting by professional technicians. In the future, machine learning algorithms should be integrated into single-molecule biosensing platforms to greatly improve the efficiency of data analysis. (4) The development of single-molecule fluorescent biosensors with the capability of simultaneously detecting a greater number of targets is highly required. (5) Monitoring intracellular disease biomarkers in real-time is essential for the study of pathological and physiological processes. The reported biosensors mainly rely on nucleic acid probes and small molecule fluorescent probes, but they are easily degraded intracellularly. The development of efficient in situ imaging strategies for single-molecule fluorescent biosensors is highly desired. (6) Single molecule fluorescent biosensors are usually demonstrated for the proof-of-concept in the lab stage. Great efforts should be put into translating these technologies into practical applications in early clinic diagnosis. The potential applications of single molecule biosensors in fundamental biomedical research and practical clinical diagnostics are expected to significantly expand in the near future, paving the way for advancements in diagnostics, drug discovery, and personalized medicine.

## Figures and Tables

**Table 1:** Comparison of single-molecule fluorescent biosensor with other biosensors for DNA assay.

Methods	Analyte Type	Linear Range	LOD	Sample Volume	Ref.
Single-molecule fluorescent-based biosensor	HPV16 DNA	1 aM–1 fM	3.0 aM	1 uL	[26]
MoS_2_/Graphene nanostructure field effect transistor-based detection	DNA	10 aM–100 pM	10 aM	N/A	[27]
Fiber-based surface plasmon resonance biosensor	secY DNA	1.0 fM–1.0 pM	1.0 fM	N/A	[28]
silica nanoparticle-enhanced microcantilever sensor	Hepatitis B Virus DNA	23.1 fM–2.3 nM	2.3 fM	20 uL	[29]
nanomechanical resonator biosensor based on a photonic crystal nanowire array	DNA	500 aM–10 pM	500 aM	1 mL	[30]
quartz crystal microbalance-based detection	DNA	1.0 fM–10 pM	0.7 fM	30 uL	[31]

**Table 2:** Summary of single-molecule biosensors for quantitative analysis of tumor biomarkers.

Target	Combined Amplification Strategy	Assay Time	Linear Range	LOD	Real Sample	Ref.
DNA	no	10 min	1 aM–1 fM	5.00 aM	vaginal secretions; MRSA-spiked milk samples	[26]
Retroviral DNA	no	70 min	100 aM–1 nM	66.1 aM, 82.8 aM	HuT-78 cells; U266B1 cells	[22]
SNP	T7 transcription	1 h	0.1 aM–100 pM	0.0724 × 10 aM; 0.0372 × 10 aM	PANC-1 cells; HCT-116 cells; HL-7702 cells; H358 cells; HLF-1 cells; and A549 cells	[59]
DNA methylation	T7 transcription	4.5 h	1 fM–10 nM	337 aM	A549 cells; HepG2 cells; HT-29 cells; HBE cells; H358 cell; breast cancer patient tissue; healthy individual tissue.	[65]
6mdA-DNA; 4mdC-DNA	TMSD	1 h	10 fM–100 nM	9.80 fM; 9.97 fM	HepG2 cells; E. coli plasmid cloning vector (pUC19)	[71]
8-oxo-7, 8-dihydroguanine		70 min	0.5 aM–5 pM	2.45 aM	A549 cells; HeLa cells; HL-7702 cells	[81]
lncRNA	PER; SDA	40 min	100 aM–1 nM	65.25 aM	MCF-7 cells; HeLa cells; A549 cells; HBE cells; breast cancer patient tissues; healthy individual tissues	[102]
MALAT1; HOTAIR	No	1 h	0.1 aM–1 pM	0.10 aM; 0.031 aM	SW480 cells; MCF-7 cells; A549 cells; HBE cells	[89]
microRNA	No	~3 h	1 fM–100 pM	1.12 fM	Serum	[100]
microRNA	PER		1 fM–100 pM	0.45 fM	H460 cells; H292 cells; H358 cells; A549 cells; and H1975 cells; NSCLC patient tissue and healthy individual tissue	[101]
Human telomerase RNA	No	1 h	10 fM–1 nM	2.10 fM	MCF-7 cells; HL-7702 cells; A549 cells; HeLa cells; breast cancer patient tissue; healthy individual tissue	[106]
piRNA	Loop connection	~5 h	1 fM–10 nM	0.104 fM	MCF-7 cells; A549 cells; HepG2 cells; HeLa cells; HCT-116 cells; breast cancer patient tissue; healthy individual tissue	[111]
circRNAs	EXPAR, RCA	~3 h	100 aM–10 pM	41.3 aM	MCF-7 cells; HepG2 cells; HeLa cells; HL-7702 cells; A549 cells; breast cancer patient tissue; healthy individual tissue	[120]
UDG	SDA	~2.5 h	0.0005–0.5 U/mL	0.00029 U/mL	HeLa cells; HL-7702 cells	[138]
hSMUG1; hAAG	SP-SDA	~4 h	1.00 × 10^−9^–0.02 U/μL	8.14 × 10^−10^ U/μL; 4.50 × 10^−9^ U/μL	A549 cells; HEK-293 cells; HeLa cells	[128]
FTO	RCA	~5 h	1 fM–1 nM	0.596 fM	MDA-MB-231 cells; MCF-10A cells; MCF-7 cells; A549 cells; HeLa cells; breast cancer patient tissue; healthy individual tissue	[121]
APOBEC3A	ERA	~3 h	1 fM–50 pM	0.855 aM	A549 cells; HEK-293 cells; HeLa cells; MCF-7 cells	[144]
telomerase	EDC	100 min	no	no	MCF-7 cells; HeLa cells; HL-7702 cells A549 cells;	[145]
ALP	HCR	~2.5 h	1 × 10^−5^–1 × 10^−2^ U/mL	2.61× 10^−6^ U/mL	HeLa cells; MCF-7 cells; HEK cells; clinical serum samples	[156]
caspase-8; caspase-9	Exo III-mediated amplification	~2.5 h	2.50 × 10^−6^ –2.50 × 10^−3^ U/μL	2.08 × 10^−6^ U/μL; 1.71 × 10^−6^ U/μL	HeLa cells; MCF-7 cells; Jurkat cells	[166]
FEN1	RCA	2.5 h	0–0.5 U/μL	2.24 × 10^−5^ U/μL	A549 cells; HeLa cells; MCF-7 cells; HepG2 cells; HL-7702 cells; HEK-293 cells; breast cancer patient tissue; healthy individual tissue	[173]
